# Retrospective Toxicological Profiling of Radium-223 Dichloride for the Treatment of Bone Metastases in Prostate Cancer Using Adverse Event Data

**DOI:** 10.3390/medicina55050149

**Published:** 2019-05-16

**Authors:** Theodoros G. Soldatos, Ioannis Iakovou, Christos Sachpekidis

**Affiliations:** 1 Molecular Health GmbH, 69115 Heidelberg, Germany; 2 Department of Nuclear Medicine, Aristotle University of Thessaloniki, Papageorgiou Hospital, 56429 Thessaloniki, Greece; iiakovou@icloud.com (I.I.); christos_saxpe@yahoo.gr (C.S.); 3 Department of Nuclear Medicine, Inselspital, Bern University Hospital, University of Bern, 3010 Bern, Switzerland

**Keywords:** prostate cancer, bone metastases, radium-223 dichloride (Xofigo^®^), side effects, real world data, data mining, pharmacoepidemiology, proportional reporting ratio

## Abstract

*Background and Objective*: Radium-223 dichloride (Xofigo^®^) is a calcium mimetic agent approved for the treatment of castration-resistant prostate cancer patients with symptomatic bone metastases and no known visceral metastatic disease. This targeted, α-particle-emitting therapy has demonstrated significant survival benefit accompanied by a favorable safety profile. Nevertheless, recent evidence suggests that its combined use with abiraterone and prednisone/prednisolone may be associated with increased risk of death and fractures. While the precise pathophysiologic mechanisms of these events are not yet clear, collecting evidence from more clinical trials and translational studies is necessary. The aim of our present study is to assess whether accessible sources of patient outcome data can help gain additional clinical insights to radium-223 dichloride’s safety profile. *Materials and Methods*: We performed a retrospective analysis of cases extracted from the FDA Adverse Event Reporting System and characterized side effect occurrence by using reporting ratios. *Results*: A total of ~1500 prostate cancer patients treated with radium-223 dichloride was identified, and side effects reported with the use of radium-223 dichloride alone or in combination with other therapeutic agents were extracted. Our analysis demonstrates that radium-223 dichloride may often come with hematological-related reactions, and that, when administered together with other drugs, its safety profile may differ. *Conclusions*: While more prospective studies are needed to fully characterize the toxicological profile of radium-223 dichloride, the present work constitutes perhaps the first effort to examine its safety when administered alone and in combination with other agents based on computational evidence from public real-world post marketing data.

## 1. Introduction

Development of bone metastases represents a pivotal incident in the clinical course of prostate cancer (PC), associated with the appearance of skeletal-related events, a decrease in quality of life and an increase in mortality [1,2]. The skeleton is the major metastatic site in PC with more than 90% of patients dying from PC having bone metastases [3]. Several classes of bone-targeting therapies have been developed and introduced in clinical practice for the treatment of PC-related bone metastases, some of which have a proven capacity to improve survival, and others with rather supportive or palliative role.

Historically, radionuclide therapy has been applied for decades in the treatment of bone metastases with bone-modifying, β-particle-emitting agents (like strontium-89 and samarium-153), but their use has been mainly palliative and without clear survival benefit when applied as monotherapy [2]. Unlike previous radiopharmaceuticals, radium-223 dichloride (Xofigo^®^) [4,5] has been the first targeted, α-particle-emitting agent to show a survival benefit in castration-resistant PC (CRPC) patients with bone metastases, as demonstrated in the landmark ALSYMPCA trial [6]. This study demonstrated an improvement of overall survival as well as delaying of time to first symptomatic skeletal event with the use of radium-223 dichloride, and led to its Food and Drug Administration (FDA) approval in 2013 for the treatment of CRPC patients with symptomatic bone metastases and no known visceral metastatic disease.

Besides its efficacy in controlling metastatic bone disease, the ALSYMPCA trial highlighted another significant feature of radium-223 dichloride, namely its favorable safety profile; ALSYMPCA’s final long-term safety analysis confirmed low myelosuppression incidence and further showed that treatment remained well tolerated, with no new safety concerns [6,7,8]. These results suggested the suitability of the agent for combined use with other medications. Initial results regarding concurrent administration of radium-223 dichloride with docetaxel or anti-androgen therapies seemed promising regarding tolerance and safety of the concomitant therapies [9,10,11]. Nevertheless, recent preliminary data of an ongoing, double-blind, placebo-controlled phase III trial in metastatic CRPC comparing radium-223 dichloride with placebo, both given in combination with abiraterone and prednisone/prednisolone, have indicated that combined use of these medications may be associated with increased risk of death and fractures [12,13,14]. Based on these results, the European Medicines Agency (EMA) has recommended that the use of the combination therapy radium-223 dichloride with abiraterone and prednisone/prednisolone should be restricted [12,13,14]. Safety-related warning and precautionary information was also accordingly updated in radium-223 dichloride’s FDA label [15].

Inspired by these developments and the importance of radium-223 dichloride in the treatment of PC patients, we searched for sources of accessible real-world data and attempted to characterize its safety profile. Specifically, we analyzed adverse events (AEs) from the FDA’s Adverse Event Reporting System (FAERS) and extracted side effects reported in PC patients treated with radium-223 dichloride alone or in combination with other therapeutic agents. Last, we share our findings with the community to support the current efforts to characterize evidence regarding radium-223 dichloride’s safety.

## 2. Materials and Methods

For the side effect profiling of radium-223 dichloride we relied on the analysis of 7.9 million AEs extracted from the public FAERS dataset (incl. 2017Q2).

### 2.1. Data Integration

The dataset held information regarding patients’ treatments (medications) and indications (disease or condition), as well as the reported reactions and outcomes (e.g., “death” or “hospitalization”) observed in AEs. To compensate for ambiguities introduced by the non-standardized use of drug names [16], medications reported via free text in FAERS were consolidated and via a stepwise process matched to standardized dictionaries [17]. This drug-centric integration process allowed drugs to be further categorized according to the Anatomical Therapeutic Chemical (ATC) classification system. Indications and reactions, coded by FAERS in terms from the Medical Dictionary for Regulatory Activities (MedDRA), were further contextualized by using its full hierarchical structure.

### 2.2. Statistical analysis

Using this dataset, we could then define two AE cohorts:

- ***Cohort A***: 1021 patients treated with radium-223 dichloride alone.
- ***Cohort B***: 542 patients treated not only with radium-223 dichloride, but also with other drugs.

For the statistical characterization of the two cohorts, we employed the proportional reporting ratio (PRR) metric, using the approach described by van Puijenbroek et al. [18]. PRR is an established measure of disproportionality in pharmacovigilance and a valuable statistical aid to the evaluation of signals generated from spontaneous reporting data [19]. Each cohort was characterized with respect to the occurrence of drugs, serious outcomes, and indications and reactions. We also considered relevant statistical significance be reflected by Fisher’s exact test *p*-values (two-tailed). 

Results for each cohort A and B are summarized in the corresponding Supplementary files A and B, respectively. The supplementary files also contain drug descriptions in terms from the different levels of the ATC categorization, and indications and reactions are accordingly described in terms from the different levels of the MedDRA hierarchy. Results per cohort list the observed case counts (i.e., number of AEs a certain occurrence was observed in), percentage of cohort cases, the PRR disproportionality score, and Fisher’s test *p*-values. The Supplementary files contain for consideration by the community all observations irrespective of their PRR or Fisher’s exact test signals. For this study, we considered a *p*-value of 5% or lower to indicate statistical significance (i.e., when *p*-value < 0.05).

## 3. Results

In total, 1563 AEs involving radium-223 dichloride therapy were identified: 1021 cases reported treatment with radium-223 dichloride only (***Cohort A***), whereas in the remaining 542 cases patients were treated with other drugs as well (***Cohort B***). Of the total radium-223 dichloride set of AEs, 1121 cases explicitly linked to an indication, 1027 of which (91.6%) confirmed use in PC patients. We manually examined the comorbidities mentioned in the set and found that they included primarily metastatic (bone) disease. Considering that radium-223 dichloride is specifically approved for the treatment of CRPC patients, it was concluded that the identified AE cases adequately reflect its use in clinical practice. We therefore decided to investigate the whole set of 1563 AEs and keep the pool of side effect observations as broad as possible.

Moreover, we examined and confirmed that drugs co-medicated with radium-223 dichloride in Cohort B (apart from Xofigo^®^, another 444 drug records matched in this AE set) properly reflect the oncological clinical practice in CRPC with osseous metastases. Specifically, the most frequently co-medicated category was anti-androgens, reported in 197 AEs of this set (i.e., 36.35% of those cases, and with the highest PRR signal 94.84), while other frequently co-administered treatment categories included glucocorticoids, opioids and other analgesics, as well as bisphosphonates.

### 3.1. Outcome Analysis

We examined serious outcomes reported in radium-223 dichloride’s AEs—Table 1 lists percentage (%) of cases per cohort that have the respective outcome reported. Overall, patients treated with radium-223 dichloride (cohort noted as ‘A ∪ B’ in Table 1) had somewhat less occurrence of Death, Life threatening and Hospitalization cases than reported for the average PC or cancer patient in FAERS. In addition, we noticed that cohort B patients tend to suffer worse outcomes than in cohort A. This likely reflects a situation in which cohort B patients—treated with multiple drugs, rather than with radium-223 dichloride alone—may have experienced more complicated disease conditions (e.g., larger tumor burden or multiple comorbidities). Nonetheless, no definitive conclusions can be drawn directly from the present dataset alone.

### 3.2. Side Effect Profiling

Next, we compared the occurrence of reactions mentioned in each cohort A and B. We examined reactions at the Preferred Term (PT) level of MedDRA (level 4 categories), and excluded from the analysis terms that reflected tumor/staging status (e.g., PC, disease progression, metastasis to bone), that were unspecific (e.g., laboratory test abnormal, pain) or that did not represent drug-induced effects (e.g., underdose, drug ineffective). Table 2 summarizes the twenty such most frequently reported reactions in each cohort (see also Supplementary files A and B). The table juxtaposes the relative order (#Rank) of each reaction per cohort based on its frequency in that set, the number of cases (AEs) and percentage of that cohort’s AEs (%Set) that had the respective reaction reported, and the corresponding PRR score per cohort. Our FAERS analysis could recapitulate several known side effects listed in radium-223 dichloride’s label. Overall, cohort A had fewer side effects reported than cohort B, despite its larger patient population. In specific, a total of 598 MedDRA PTs (level 4 terms) were linked to cohort B whereas, in comparison, 499 were described in cohort A.

Furthermore, hematological side effects had strong signals for both cohorts (examples mentioned next refer all to statistically significant observations per set, with *p*-value <0.05):

- **General hematotoxicity reactions** include examples such as the *PT Blood count abnormal* with strong signal for both cohorts A (3.23%, PRR = 43.82) and B (1.11%, PRR = 14.94), as well as the term *Bone marrow failure* (1.76%, PRR = 12.42) for Set A.
- **Red blood cell reactions** include examples such as *Haemoglobin decreased* (Set A: 7.15%, PRR = 11.07; Set B: 3.69%, PRR = 5.71) or *Anaemia* (Set A: 3.43%, PRR = 2.98; Set B: 11.99%, PRR = 10.44).
- **White blood cell reactions** include terms like *White blood cell count decreased* (Set A: 1.86%, PRR = 3.48; Set B: 1.85%, PRR = 3.45) or *Leukopenia* (Set B: 2.21%, PRR = 8.39).
- **Thrombocytopenia** (Set A: 2.84%, PRR=4.8; Set B: 5.54%, PRR = 9.36) or *Platelet count decreased* (Set A: 4.21%, PRR = 7.52; Set B: 4.8%, PRR = 8.57).
- **Neutropenia** (Set B: 1.29%, PRR = 2.36), *Febrile neutropenia* (Set B: 1.29%, PRR = 4.24), or *Neutrophil count decreased* (Set A: 1.18%, PRR = 6.5).
- **Pancytopenia** (Set A: 1.96%, PRR = 5.99; Set B: 4.98%, PRR = 15.24)

In line with the known toxicological profile of the agent [4,5], we found that gastrointestinal effects (such as nausea, diarrhea and vomiting) were frequently reported. The list of the top most reported reactions (Table 2) also included effects reflecting a general health deterioration status (such as malaise, fatigue and asthenia) as well as reactions that referred to nutrition (e.g., decreased appetite and weight), bone and back pain events. Regarding other known side effects of radium-223 dichloride, we found that it was not easy to clearly determine their extent. For example, while the signal for some types of injection site reactions could be captured at the level of more general MedDRA categories (e.g., MedDRA HLT ‘Oedema NEC’ linked to 1.86% and 3.14% of cohorts A and B, respectively), others could not be further summarized because they had either too few occurrences or could only be expressed at more detailed MedDRA levels (e.g., erythema). The full characterization of Cohorts A and B can be found in Supplementary files A and B, respectively.

## 4. Discussion

In an attempt to investigate the side effects of radium-223 dichloride, applied alone or in combination with other therapeutic agents, we examined respective reaction occurrence in AEs extracted from public FAERS data. FAERS contains valuable AE information for a large number of patients (7.9 million cases) coming directly from healthcare professionals, consumers, and manufacturers. Our results are thus based on real world events and aim to provide additional insight to previous and current radium-223 dichloride safety profiling efforts.

Overall, we found that the larger cohort A (in which patients were treated only with radium-223 dichloride) had fewer side effects reported than the smaller Cohort B (in which patient therapy included additional treatments). While this may be somewhat expected due to the effects that the other drugs may introduce, looking at the data alone cannot provide a causative explanation. In addition, the variability between the two cohorts’ profiles may be attributable to their relative size difference, to the co-medications’ own side effects, and also to potential combinatorial therapy results. One such example is *osteonecrosis*, an effect known to be caused when radium-223 dichloride therapy is combined with (current or prior) bisphosphonate treatment (osteonecrosis of the jaw) or in patients under a long-term treatment with glucocorticoids [4,20]. Also, chemotherapy may affect myelosuppression incidence [21], making it one of the major concerns regarding radium-223 dichloride administration and its principal side effect [4,5].

Indeed, our analysis confirms the manifestation of known radium-223 dichloride side effects such as hematotoxicity; specifically, anemia, thrombocytopenia, neutropenia and bone marrow toxicity were some of the most frequently reported reactions in radium-223 dichloride’s AEs. In line with its toxicological profile, gastrointestinal disorders (such as diarrhea, vomiting, and nausea) during radium-223 dichloride treatment were also confirmed. These events can be of particular significance with respect to patient management since they may lead to dehydration, thus requiring careful monitoring of patient oral intake and fluid status [5].

Such implications also highlight the importance of computationally analyzing real world and big data towards their translation into more informed patient management [22,23,24,25]. For example, the high bone and/or back pain signals observed in radium-223 dichloride AEs may likely be attributed to progression of skeletal disease burden. However, physicians should consider also the possibility that patient symptomatology is, among others, due to a seldom described clinical *flare* phenomenon caused by the treatment, or other events such as spinal cord compression or fractures [26].

Characteristically, regulatory authorities have recently posed concerns regarding the incidence of fractures when radium-223 dichloride is combined with abiraterone and prednisone/prednisolone [14]. In our dataset, occurrence of fractures was the same for both cohorts A and B. Specifically, the High Level Group Term (HLGT) of MedDRA (level 2 category) *fractures* linked to 2.7% of both sets’ cases—namely, to 28 and 15 AEs of cohorts A and B, respectively. Interestingly, radium-223 dichloride was co-medicated with anti-androgens in five out of the fifteen AEs of Cohort B that reported fractures. Nevertheless, we expect that deriving conclusive hypotheses on the existence of potential synergistic effects between these agents would require examining more data gathered from additional studies in this context.

Previous studies report that abiraterone’s mechanism of action involves suppression of androgen production by blocking the enzyme activity of Cytochrome P450 17 α-hydroxylase (CYP17), providing an inhibitory effect on CRPC progression [27]. However, both radiation therapy and androgen receptor-directed therapy can induce significant oxidative stress through an increase of reactive oxygen species, potentially causing various side effects. Moreover, androgen receptor-directed therapies can induce hormonal imbalance with induction of glucocorticoid receptor expression in resistant CRPC clones [28,29]. Thus, the combination treatment of radium-223 dichloride, abiraterone and prednisone/prednisolone could potentially exacerbate the toxicity issue raised by the recently published preliminary data of the phase III trial in metastatic CRPC [12,13,14]. However, from FAERS data only it cannot be derived which are the potential mechanisms underlying the effects of combinatorial therapy on the toxicological profile of radium-223 dichloride, emphasizing thus in addition the importance of being able to molecularly analyze real world AE data coming from spontaneous reporting systems [17,30,31].

Moreover, AE data may also come with other limitations [17,22,23]. For example, the severity of conditions (indications, reactions) reported in FAERS is not graded and their occurrence cannot be confirmed. In addition, the public FAERS dataset may include reporting errors; it also does not adequately suffice to clearly determine whether some of the observed reactions reflect disease symptoms and patient conditions or not. The high occurrence of reactions representing a general health deterioration status (such as malaise, fatigue and asthenia) in radium-223 dichloride AEs may thus indicate side effects caused by the treatment but may also reflect signs of disease grade and/or tumor progression. Furthermore, FAERS contains only AEs and is therefore biased without proper normalization considering reference/control data. In turn, reaction occurrence in our dataset may differ from radium-223 dichloride’s product characteristics summary [4,5]. Last, public FAERS does not come with (potentially important) information regarding a patient’s history (e.g., therapies, allergies, co-morbidities) prior to their AE.

Furthermore, one other important parameter regarding the use of radiopharmaceuticals is dosimetry. At present, radium-223 dichloride is applied according to standard fixed administrations at 4-week intervals, modified according to patient weight. However, therapy individualization and optimization would also involve internal dosimetry calculations. Although the nature of α-irradiation in a clinical context is not clearly understood [32], different pharmacokinetic, biodistribution and dosimetry studies have demonstrated a rapid clearance of radium-223 from the blood with the gut being the main route of excretion [33,34], partially explaining the high incidence of gastrointestinal track side-effects. Regarding bone marrow toxicity, it is not expected that uptake of radium-223 on bone surfaces will irradiate the marrow cavities uniformly, due to the high linear energy transfer and short path length of α-particles [32]. Interestingly, a spatial gradient of hematopoietic stem and progenitor cells has been demonstrated within human cancellous bone with higher concentrations near the bone surfaces. The dosimetric implication of this finding is significant in terms of radium-223 dichloride treatment, in which the absorbed dose is non-uniformly delivered across the bone marrow, leading to higher absorbed doses in these radiosensitive cells of interest [35]. On the other hand, patient dosimetry data in tumor lesions, which could potentially lead to an increase of the radiopharmaceutical dose administered without increasing the incidence of side effects, are limited. Moreover, despite being feasible, the clinical benefit of tumor macrodosimetry of radium-223 remains to be investigated [36].

Finally, our AE analysis could benefit further from the examination of additional data regarding laboratory and clinical parameters (e.g., baseline hemoglobin values, number of radium-223 dichloride injections and dosimetry, treatment duration, cycles, and dosage). However, such data are not readily available or straightforward to extract from the public FAERS dataset. Nonetheless, FAERS reports contain a large breadth of drug-induced phenotypic effects observed in AEs that can be further analyzed by capitalizing upon its integration with additional levels of information [17,31]. Also, AEs comprise an augmented data stream capturing real-world scenarios regarding therapeutic uses and combinations, phenotypes and conditions not studied in clinical-trials, as well as include information for many more patients [17,30]. Therefore, with our work, we also highlight the importance of standardizing and structuring real world data and invite the development of more systematic approaches that strive to efficiently combine outcome data with molecular etiologies and clinically significant information. Last, we envisage that our findings will provide additional context to the current efforts to characterize evidence regarding the safety of radium-223 dichloride.

## 5. Conclusions

Radium-223 dichloride (Xofigo^®^) is a radiopharmaceutical approved for the treatment of CRPC patients with symptomatic bone metastases and no known visceral metastatic disease. Despite its favorable safety profile, recent evidence suggests that its combined use with abiraterone and prednisone/prednisolone may be associated with increased fracture and mortality risk. While our results acknowledge these concerns posed by the regulatory authorities (EMA, FDA), we find that deriving definitive hypotheses on this aspect from our AE data would require examining a larger sample of patients and we call for more studies to help gather additional data regarding the combined application of these agents. Overall, our results recapitulate known side effects observed with radium-223 dichloride and confirm hematotoxicity and gastrointestinal disorders as the main patient safety concerns. While public FAERS-based analytics might benefit from the consideration of additional clinical information, our study emphasizes the importance of computationally analyzing patient outcome information so as to support the gain of clinical insight directly from real world data.

## Figures and Tables

**Table 1:** Selected serious outcomes reported in AEs.

Outcome	Cohort (%)					
	A	B	A ∪ B ^1^	PC ^2^	Cancer ^3^	FAERS
Death	18.81	23.43	20.41	26.35	24.71	10.11
Life threatening	1.76	5.17	2.94	3.22	5.02	3.03
Hospitalization	23.99	48.71	32.57	30.15	35.37 ^1^	24.36

^1^ This cohort refers to the union of the patients included in sets A and B, namely all 1563 patients treated with radium-223 dichloride. ^2^ Prostate cancer (PC) cases were defined as those AEs that have indications linked to the High Level Term (HLT) of MedDRA (3rd level category) named *Prostatic neoplasms malignant.* ^3^ *Cancer* AEs were defined as cases with indications linked to the System Organ Class (SOC) of MedDRA (level 1 category) called *Neoplasms benign, malignant and unspecified (incl. cysts and polyps)*.

**Table 2:** Most frequently reported side effects per cohort.

Side Effect (Reaction)	Cohort A: Only Xofigo				Cohort B: Xofigo and Other Drugs			
Name	#Rank	AEs	%Set	PRR	#Rank	AEs	%Set	PRR
Haemoglobin decreased ^1^	1	73	7.14985	11.07178	16	20	3.69004	5.7086
Nausea	2 ^+^	51	4.9951	1.16074	3	64	11.80812	2.74418
Platelet count decreased ^2^	3	43	4.21156	7.52259	14	26	4.79705	8.56563
Malaise	4	39	3.81978	1.56369	15	21	3.87454	1.58605
Diarrhoea	5 ^+^	38	3.72184	1.25585	4	50	9.22509	3.11315
Anaemia ^1^	6	35	3.42801	2.98228	2	65	11.99262	10.43733
Bone pain	7	35	3.42801	10.41557	7	32	5.90406	17.93774
Vomiting	8 ^+^	34	3.33007	1.27435	8	30	5.53506	2.11824
Blood count abnormal ^3^	9	33	3.23213	43.82126	>20	6	1.10701	14.94108
Fatigue	10 ^+^	31	3.03624	0.83291	1	90	16.60517	4.5564
Thrombocytopenia ^2^	11	29	2.84035	4.80398	9	30	5.53506	9.36238
Asthenia	12 ^+^	25	2.44858	1.17253	6	41	7.56458	3.62295
Pneumonia	13 ^+^	21	2.05681	1.27006	>20	17	3.13653	1.93684
Pancytopenia	14	20	1.95886	5.98948	11	27	4.98155	15.23674
White blood cell count decreased	15	19	1.86092	3.48364	>20	10	1.84502	3.45335
Bone marrow failure ^3^	16	18	1.76298	12.42202	>20 ^+^	5	0.92251	6.49296
Decreased appetite	17 ^+^	17	1.66503	1.31554	5	45	8.30258	6.56207
Back pain	18 ^+^	15	1.46915	1.13896	10	28	5.16605	4.00575
Weight decreased	19 ^+^	14	1.3712	0.949	12	27	4.98155	3.44827
Neutrophil count decreased	20	12	1.17532	6.49458	>20 ^+^	3	0.55351	3.05684
Pyrexia	>20 ^+^	13	1.27326	0.65169	19	18	3.32103	1.69995
Dyspnoea	>20 ^+^	9	0.88149	0.28814	13	26	4.79705	1.56826
Osteonecrosis	>20 ^+^	9	0.88149	2.34167	17	19	3.50554	9.31611
Arthralgia	>20 ^+^	8	0.78355	0.40463	18	19	3.50554	1.81054
Headache	>20 ^+^	5	0.48972	0.14564	20	18	3.32103	0.98778

^1^ The ‘Haemoglobin decreased’ and ‘Anaemia’ terms refer to the same condition. ^2^ The terms ‘Platelet count decreased’ and ‘Thrombocytopenia’ refer to the same condition. ^3^ ‘Blood count abnormal’ and ‘Bone marrow failure’ refer to the same condition. ^+^ Reactions occurring in less than 1% of the respective cohort’s cases, that were found to not be statistically significant (*p*-value > = 0.05), or both.

## Supplementary Materials

Supplementary materials can be accessed at: https://www.mdpi.com/1010-660X/55/5/149/s1.
